# Firearm Homicide Demographics Before and After the COVID-19 Pandemic

**DOI:** 10.1001/jamanetworkopen.2024.12946

**Published:** 2024-05-22

**Authors:** Alex R. Piquero, John K. Roman

**Affiliations:** 1 Department of Sociology and Criminology, University of Miami, Coral Gables, Florida; 2 NORC at the University of Chicago, Illinois

## Abstract

This cross-sectional study evaluates changes in firearm homicide demographics before and after the COVID-19 pandemic.

## Introduction

In 2020, the US experienced the largest 1-year increase in homicide since 1960. The spike began in the first few months of the year, accelerating during the COVID-19 pandemic, emergency measures, the murder of George Floyd, and social protests.^1^

Three additional observations are relevant. First, the US Centers for Disease Control and Prevention (CDC) reported that the homicide increase in 2020 was due to firearm injuries. While the overall homicide rate increased 28.4%, the firearm homicide rate increased 34.6%.^2^ Second, the spike in violence was concentrated within certain demographic groups. CDC researchers found 19 384 victims of firearms homicide in 2020.^3^ Of those victims, 61% were Black individuals, and they experienced firearm homicide at 14 times the rate of White indviduals in 2020. This racial disparity does not exist for other types of violence.^4^ Third, the largest increases in death by firearm homicide were for Black men aged between 10 and 44 years old.^5^

## Methods

This cross-sectional study queried mortality data from the CDC WONDER online database to examine race and ethnicity disparities in death by firearm homicide by 5-year age-band categories for the period 2018 through 2022 (2022 includes provisional data), before, during, and subsequent to the pandemic.^6^ The CDC WONDER database provides information on race and ethnicity as reported on death certificates. Population-adjusted rates were returned by the database using data from the US Census Bureau. Exemption of institutional review board review was granted by The University of Miami. Informed consent was not required because the study used publicly available data without personal identifying information. All analyses were completed in Excel version 16.0 (Microsoft).

## Results

**Figure 1.**
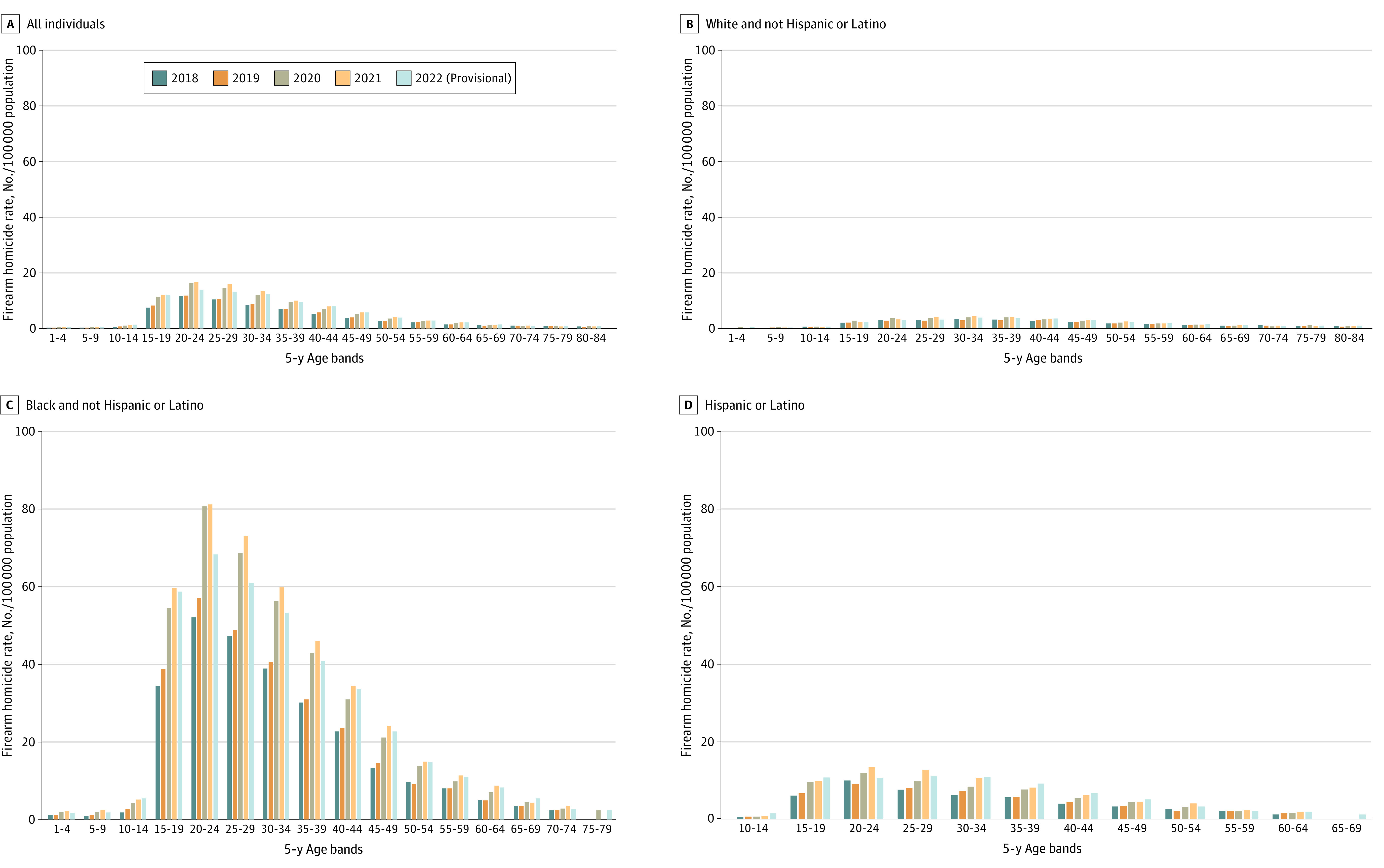
Firearm Homicide Victimization Crude Rates

**Figure 2.**
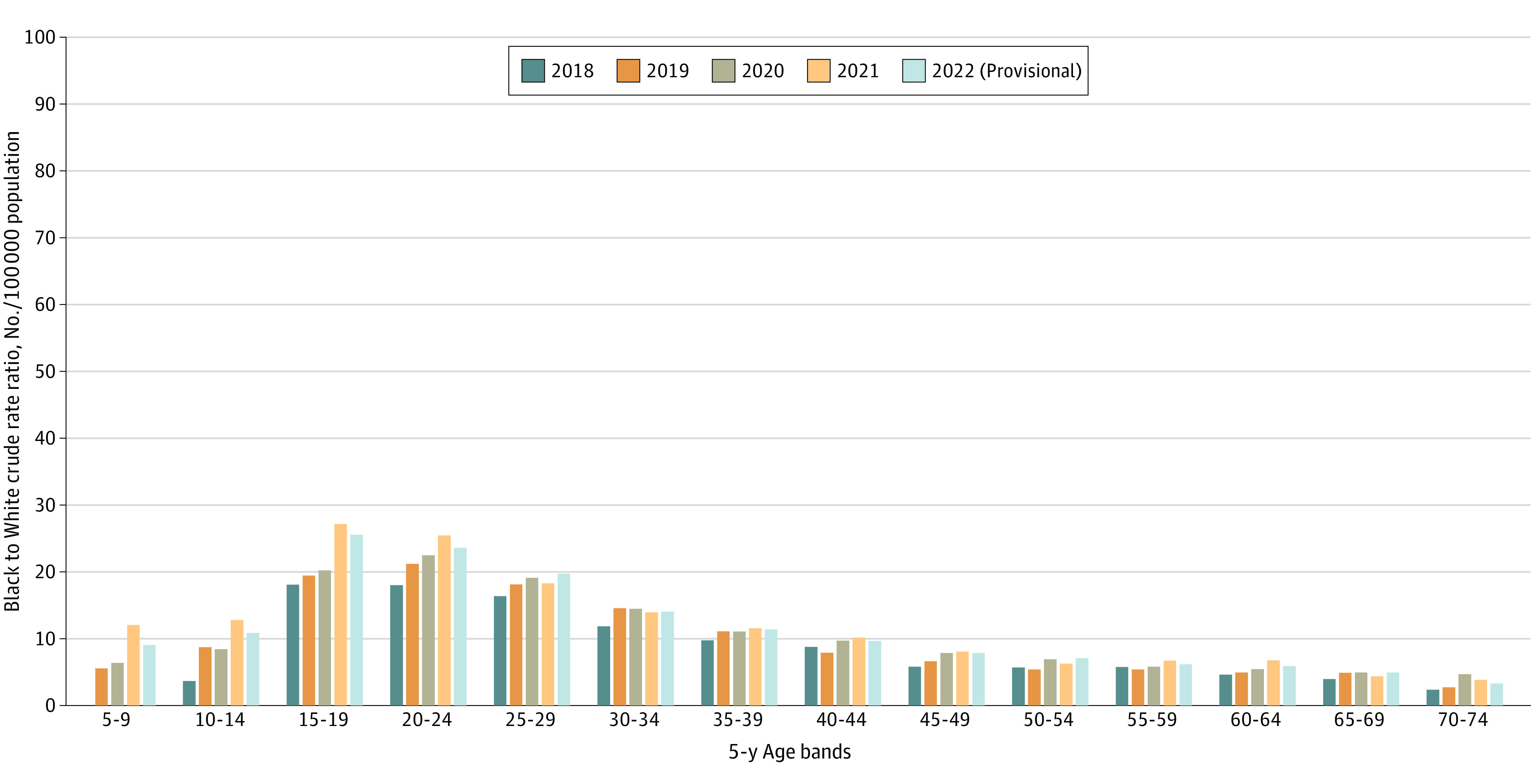
Ratio of Black to White (Non-Hispanic or Latino) Individuals’ Firearm Homicide Victimization Crude Rates

Figure 1 displays the crude rate of death by firearm homicide per 100 000 for all individuals, White individuals only, Black individuals only, and Hispanic or Latino individuals of any race. Four results are evident. First, among all groups, 2 age bands (20-24 and 25-29 years) show the highest peaks. Second, the year 2021 has the highest rate for the majority of age bands. Third, firearm homicides for White individuals also peaked in 2021, with persons aged 20 to 34 years having the highest risk. Fourth, rates for White individuals never top 4.3 per 100 000. Among Black individuals, for those persons aged 20 to 24 years, the rate is over 80 per 100 000. Finally, Figure 2 displays the ratio of Black to White homicide victimization by age group, by year. For those aged 15 to 19 years, the rate for Black individuals in 2021 is 27 times the rate for White individuals.

## Discussion

In this study, death by firearm homicide was concentrated among Black individuals aged 15 to 24 years before, during, and subsequent to the COVID-19 pandemic, implying that there are likely to be social and structural conditions that contribute to these racial disparities. This study is limited because of its focus solely on firearm homicides. Study findings have implications for prevention and intervention strategies addressing the needs of those persons at highest risk and must consider short- and long-term strategies involving law enforcement, community groups, education, and health care professionals.

## Notes

Supplement.
Data Sharing Statement
