# Supplementary material for: Firearm Homicide Demographics Before and After the COVID-19 Pandemic
Source: JAMA Netw Open. 2024 May 22;7(5):e2412946. doi: 10.1001/jamanetworkopen.2024.12946 (PMC11112446; doi:10.1001/jamanetworkopen.2024.12946)
Supplement: Supplement. — Data Sharing Statement [file jamanetwopen-e2412946-s001.pdf]

Data Sharing Statement

Piquero. Firearm Homicide Demographics Before and After the COVID-19 Pandemic. *JAMA Netw Open*. Published May 22, 2024. doi:10.1001/jamanetworkopen.2024.12946

Data
Data available: No

Additional Information
Explanation for why data not available: Data are publicly available from CDC.
